# Seven key parameters that facilitate clinical pharmacy practice: a comparison between Israel and the United States

**DOI:** 10.1186/s13584-021-00476-8

**Published:** 2021-06-30

**Authors:** Adam J. Rose, Daniel Witt, Carmil Azran, Ran Nissan

**Affiliations:** 1 Hebrew University School of Public Health, Jerusalem, Israel; 2 University of Utah College of Pharmacy, Department of Pharmacotherapy, Salt Lake City, UT USA; 3 Department of Clinical Pharmacy, Herzliya Medical Center, Herzliya, Israel; 4 Pharmacy Services, Beilinson Hospital, Rabin Medical Center, Petah Tikva, Israel; 5 Beit Rivka Geriatric Rehabilitation Center, Petah Tikva, Israel

**Keywords:** Clinical pharmacy, Medication therapy management, Mid-level providers, Health professions, History of medicine, International comparison of healthcare systems, Health law

## Abstract

Clinical pharmacists have advanced training that enables them to manage medication therapy, including prescribing, titrating, and discontinuing medications, in order to achieve therapeutic goals. In some countries, such as the United States, advances in training, responsibility, legal frameworks, and public acceptance of new roles have proceeded in parallel to expand the scope and contribution of clinical pharmacists over several decades. In this manuscript, we detail seven discrete key parameters of professional advancement for clinical pharmacists, corresponding to the seven areas in which they must advance in order to contribute fully to delivering high-quality medical care. For each key parameter, we briefly summarize the progress made in the United States to date, as well as goals for future progress. We then compare this to the development of the analogous key parameter in Israel. We found that on some key parameters, the development of clinical pharmacy in Israel lags behind the United States. This manuscript can provide a roadmap for the future advancement of clinical pharmacy in Israel, toward its full realization as a profession that can contribute to delivering high-quality medical care.

## Background

Clinical pharmacists have advanced training focusing on skills such as interviewing patients and directly managing medications to achieve predetermined goals. In addition, they are qualified to perform traditional tasks that any pharmacist would, including compounding, dispensing, and advising patients on the proper use of a medication. There are many highly-accepted definitions for clinical pharmacy; here, we present the official definition of the American College of Clinical Pharmacy:

The American College of Clinical Pharmacy (ACCP) defines clinical pharmacy as an area of pharmacy concerned with the science and practice of rational medication use. Clinical pharmacy is a health science discipline in which pharmacists provide patient care that optimizes medication therapy and promotes health, and disease prevention. The practice of clinical pharmacy embraces the philosophy of pharmaceutical care, blending a caring orientation with specialized therapeutic knowledge, experience, and judgment to ensure optimal patient outcomes. As a discipline, clinical pharmacy also has an obligation to contribute to the generation of new knowledge that advances health and quality of life [1].

A similar definition has been advanced by the Israeli Ministry of Health [2], suggesting a similarity of aspirational goals. Clinical pharmacists can serve multiple roles, including as advisors to other clinicians, as policy advisors, as quality officers, and as direct care providers [3]. In practice, what truly separates clinical pharmacy from more traditional pharmacy roles is playing a role as a direct provider of medical care, in partnership with physicians and other health professionals. This includes functions such as initiating, discontinuing, and titrating medications, as well as related history-taking and ordering and acting upon laboratory tests to enable effective medication management.

The purpose of this manuscript is to contrast the historical development of clinical pharmacy in Israel with that of the United States, and through this comparison to present recommendations for the future development of clinical pharmacy in Israel. In the United States, clinical pharmacy has developed an identity of its own, along with the development of seven key parameters that allow it to flourish. None of these key parameters can develop without the others; that is, they tend to advance together, or not at all. For each parameter, we look at the history of its development in the United States, as a point of comparison. Then, we compare this with the present state of affairs in Israel. For our history of the development of clinical pharmacy in the United States, we referred to the excellent ACCP report entitled, “Clinical Pharmacy in the United States: Transformation of a Profession” [4]. Those interested in reading about the historical development of clinical pharmacy in the United States in detail are urged to refer to that report; our summary of the topic is necessarily superficial and serves mainly as a point of comparison, and relies heavily on facts collected in that report.

Also, our manuscript is not the first to describe the current state of affairs in Israel with regard to the profession of clinical pharmacy [5–11]. What is new about the present manuscript is its comparative nature, and its exposition of seven discrete (but interdependent) key parameters that are necessary to support the full realization of clinical pharmacy as a profession. Our manuscript can therefore point to what changes will be needed in the future for clinical pharmacy in Israel to more fully contribute to delivering high-quality medical care.

### Defining seven key parameters

As stated above, we have defined seven key parameters whose development is necessary to allow full expression of clinical pharmacy as a profession. These parameters were defined through discussion among the four authors of this manuscript, by mutual agreement. They are:

1. Advanced Degree: Having an advanced degree for pharmacy, which is compatible with the skills required for the practice of clinical pharmacy
2. Residency: The existence of postgraduate residency programs for pharmacists, which are specifically intended to allow them to acquire and refine the skills necessary to practice as clinical pharmacists
3. Credentialing: Recognition of clinical pharmacy as a specialty within the pharmacy profession via specific credentialing
4. Supply: Having enough clinical pharmacists to perform the work demanded of them
5. Scope of Practice: Scope of practice, including legal permission to practice semi-independently in a meaningful way, prescribing medications, ordering and interpreting labs, and taking a medication-related history; relatedly, legal permission to bill for one’s work as a clinical pharmacist
6. Demand: Demand by employers (pharmacies, hospitals, clinics, managed care companies) to hire clinical pharmacists and utilize them fully
7. Acceptance: Acceptance of the clinical pharmacist role by other medical professionals as part of the healthcare team

The remainder of the paper will follow the sequence of these seven key parameters. For each, we will briefly detail its historical development in the United States and then follow with a comparison to the current situation in Israel.

### 1a) advanced degree (United States)

**Fig. 1:**
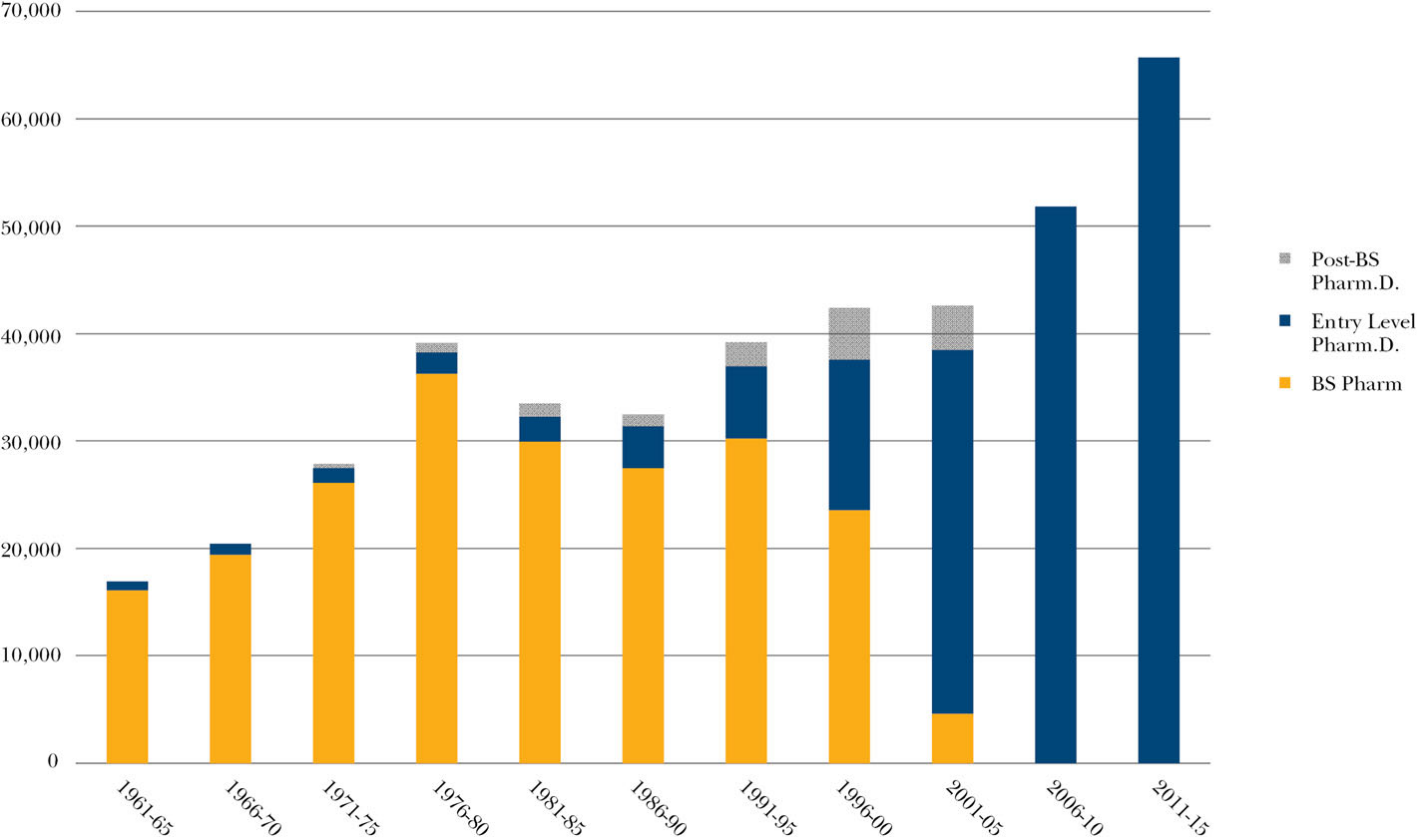
Number of Pharmacy Graduates, United States

As of 1932, pharmacy graduates in the United States universally received a 3-year Bachelor’s of Science (BS) in Pharmacy. As of 1960, this standard was increased to a 5-year BS degree. In the 1950’s, a few schools began to graduate students with a PharmD as an entry-level degree. The percentage of pharmacy graduates with a PharmD degree in the United States was small at first (Fig. 1). In the 1950’s, the pharmacy community in the United States began to discuss the idea of a PharmD as a universal entry-level degree, but due in part to concerns about imposing more educational expenses on trainees, this matter was not fully resolved until decades later. The proportion of programs offering a PharmD as an entry-level degree continued to increase throughout the 1980’s and 1990’s, reflecting a growing expectation that a PharmD would eventually be expected by future employers. By 2000, all new pharmacy students were enrolled in PharmD programs only, meaning that as of 2005 all graduates would receive a PharmD without exception. Thus, for 15 years now, all pharmacy graduates in the United States have received a PharmD [4].

### 1b) advanced degree (Israel)

In Israel, all pharmacists have at least a bachelor’s degree in pharmacy (BSc), a four-year program like other undergraduate degrees in Israel. Such a BSc degree is the minimal requirement to practice as a pharmacist in Israel and is also required as a prerequisite for enrollment in advanced pharmacy degrees. There are currently two programs in Israel that offer higher degrees in Clinical Pharmacy. The first program is at the Hebrew University of Jerusalem (HUJI) which has offered an MSc degree in clinical pharmacy since the 1980’s, and a PharmD degree as of 2013. The second program, initiated in 2017 by Ben Gurion University of the Negev (BGU), offers an MSc degree in Community Clinical Pharmacy and Regulatory Management. At present, there are 9565 active pharmacists in Israel [12], of whom approximately 150 have one of these advanced degrees and practice as clinical pharmacists, and the remainder have only the BSc [6]. Israel is a nation with a high proportion of immigrants, so some of those with a PharmD degree received it elsewhere, prior to arriving in Israel. Israel’s only PharmD program graduates 15–20 students annually, with 119 having graduated to date; there are no immediate plans for additional programs to offer a PharmD. The newer MSc program at BGU has about 30 graduates each year. Numbers of graduates to date from each program are shown in Table 1.

**Table 1:** Number of clinical pharmacy graduates in Israeli universities

Year	Hebrew University (PharmD)	Ben Gurion University (MSc)
2012	15	–
2013	8	–
2014	15	–
2015	11	–
2016	14	–
2017	11	–
2018	22	–
2019	19	30
2020	14	30

### 2a) postgraduate residency programs (United States)

**Fig. 2:**
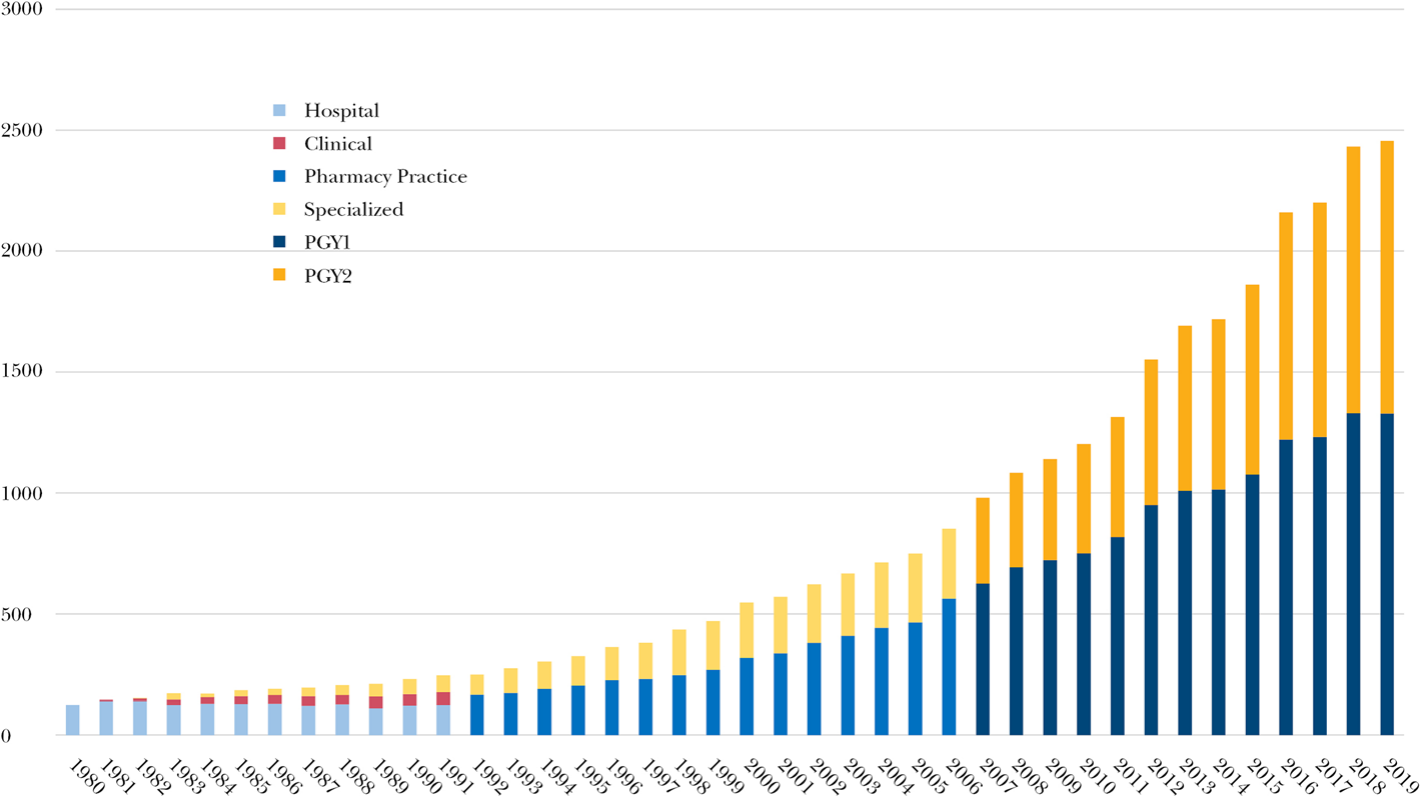
Growth in ASHP-Accredited Residency Programs 1980-2019, United States

Postgraduate residency training refers to training programs that occur after one has been granted a terminal degree, analogous to internship and residency for physicians after the MD degree has been granted. The number of postgraduate pharmacy residency programs in the United States, for pharmacists who have already received a PharmD degree, has increased precipitously since 1980, from around 100 to almost 2500 as of 2018 (Fig. 2). Residency programs usually involve practicing in a mix of both inpatient and outpatient care settings, under the supervision and tutelage of experienced clinical pharmacist faculty. The number or pharmacists completing residency training each year is now over 5000 [4]. Only those with a PharmD degree are eligible to apply for post-graduate residency programs. The first year of residency training after receiving the PharmD degree (Post Graduate Year 1, or PGY1) encompasses general principles of medication therapy management and enables the graduate to fill most positions as a clinical pharmacist in the different settings such as pharmacies, hospitals, and ambulatory-care clinics. There are also PGY1 residencies focused on other areas such as managed care. A second year of residency training (PGY 2) focuses on a specific topic, such as psychiatry, cardiology, or infectious diseases (there are currently 15 recognized specialties with PGY2 residency programs). Some PGY2 residencies focus on aspects of pharmacy management and administration, or population health. Graduates of PGY2 programs tend to seek jobs that require them to practice in this specific area, at least part of the time. In turn, an increasing number of positions require PGY1 or even specific PGY2 training. Residencies are accredited by the American Society of Health System Pharmacists (ASHP).

### 2b) postgraduate residency programs (Israel)

There are not currently any postgraduate residency programs in Israel. No employer requires such training to qualify for a particular position. However, it should be noted that the PharmD degree offered at Hebrew University includes a full year of research and two semesters of clinical rotations, with a requirement to select an area of special focus. The MSc program at BGU, meanwhile, includes a full year research thesis, as well as an adherence program that allows students a chance to interact with real patients and thus gain clinical experience. Thus, while Israel does not have residency programs per se, these degree programs do include some clinical experiences. It should also be noted, however, that PharmD programs in the United States also include similar sorts of experiences prior to granting the degree. In Israel, clinical pharmacists tend to develop specialized knowledge “on the job” through direct exposure, often without formal education or guidance. There are a number of experienced clinical pharmacists working in Israel today who would be qualified to serve as teaching faculty in residency programs if such programs were available.

### 3a) specific credentialing (United States)

**Fig. 3:**
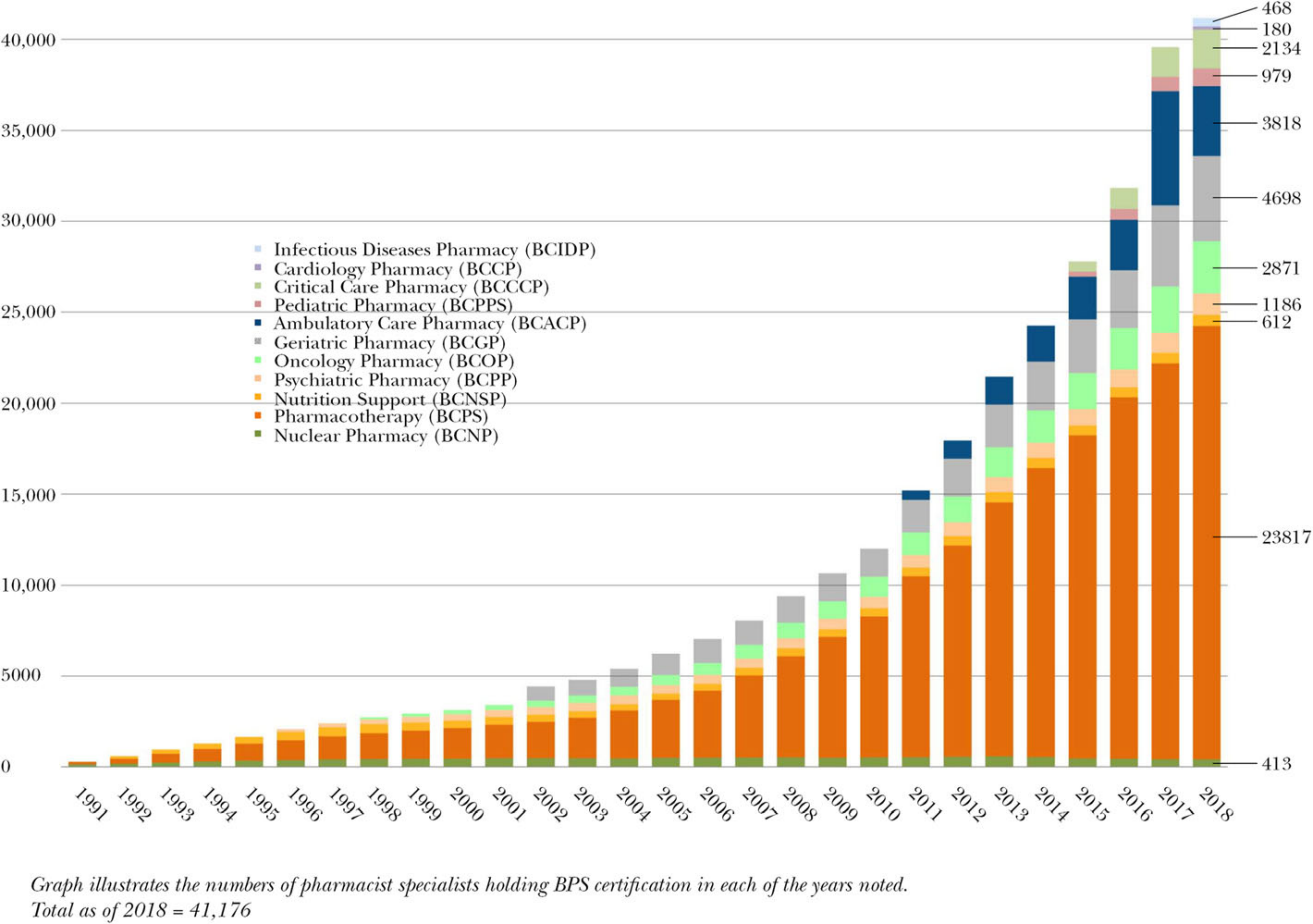
Number of Pharmacists Certified by the Board of Pharmacy Specialties, United States

In 1976, the American Board of Pharmacy Specialties (BPS) was established. BPS assessed the candidacy of clinical pharmacists to become board-certified based on their training and skills to perform this role. Candidates receive BPS certification based on meeting the qualifications for and passing a rigorous board examination, and maintain it based on continuing education or passing additional examinations. Today, an ever-increasing proportion of clinical pharmacists practicing in the United States are certified as a Board-Certified Pharmacotherapy Specialist (BCPS), or in one of the sub-specialties recognized by BPS (Fig. 3). In 2018, there were 23,817 clinical pharmacists with the general BCPS certification, and a total of 41,176 clinical pharmacists with any BPS certification, representing approximately one in eight US-based pharmacists [4]. BPS certification functions to identify those pharmacists with the requisite skills and training for high-level clinical pharmacy practice.

### 3b) specific credentialing (Israel)

In Israel, pharmacists who have completed a PharmD degree or a M.Sc. degree in clinical pharmacy are considered “clinical pharmacists” [2]. There is no equivalent of board certification for clinical pharmacists in Israel, and there are no plans to start such a program in the near future. The definition of “Clinical Pharmacy” by the Israeli Ministry of Health (MoH) does not clearly define the requirements for education and training, and there is no separate licensure for clinical pharmacists [2]. Moreover, there is no official organization or group with the authority to give pharmacy credentials, analogous to BPS in the United States. Employers themselves (such as hospitals or HMOs) are responsible for deciding which degree or qualifications will be required for which position.

### 4a) supply of clinical pharmacists (United States)

In 2019, just over 5000 individuals were accepted into the nearly 2500 pharmacy residency programs in the US. Over the last five years, residency positions have increased by 1473 positions, or 60%, nationwide [13]. This is one way of estimating the supply of pharmacists with advanced clinical training entering the US job market each year and the increasing demand for pharmacists with this skillset. As more and more pharmacists apply for PGY1 and PGY2 residencies, competition for residency positions and clinical pharmacy positions has intensified. However, clinical pharmacists are utilized in many settings. As of today, there is no shortage of clinical pharmacists, except in rural areas.

### 4b) supply of clinical pharmacists (Israel)

It is estimated that there are 250 clinical pharmacists that have advanced degrees in clinical pharmacy (M.Sc. and PharmD) [6]. Of this number, it is estimated that only 150 are active as clinical pharmacists. As recognition of clinical pharmacy grows in Israel, so does the number of positions. Based on a government report that was published in June 2016, the subset of hospitals run directly by the MoH still had 52 fewer clinical pharmacists than would be officially required based on the recommended ratio of no less than one clinical pharmacist per 100 beds [6]. Ambulatory clinics run by Israel’s four Health Maintenance Organizations (HMOs) do not have an official requirement for clinical pharmacists, although some clinical pharmacists are working in these settings as well. The Knesset Committee of Work, Welfare and Healthcare requested that the MoH submit an explanation of the planned changes that will occur in the profession of clinical pharmacy [14]. However, this report has yet to be delivered.

### 5a) scope of practice: legal permission for semi-independent practice and to receive payment for work performed (United States)

The term “semi-independent practice” means that even when performing at a high level of competence and autonomy, clinical pharmacists nevertheless must consult with physicians on some level about the most important decisions, such as whether to begin a course of therapy, or what is the treatment goal. However, while no system of care has truly proposed that clinical pharmacists should practice with full independence, systems vary widely in terms of how much authority is delegated to clinical pharmacists, and therefore how much they are able to contribute to care.

Scope of practice for clinical pharmacists encompasses the ability to perform such tasks as writing prescriptions, ordering and interpreting relevant laboratory tests, administering immunizations, and taking a medication-related history. There are many details, such as: can the clinical pharmacist only renew a previous prescription, or can they initiate new prescriptions as well? In addition, related issues include whether a clinical pharmacist can bill insurers for doing such work, and whether they can be considered the provider of record for a clinical encounter for billing purposes. These laws vary among the 50 states in the United States, with some states having more permissive rules than others. In some states, pharmacists are allowed to practice relatively independently, but not to specifically bill insurers; in others, it is the reverse. In general, these regulations have become more permissive over time, allowing pharmacist greater scope in what they can do, although at differing rates in different states. Pharmacy professional organizations continue to lobby for legal frameworks that will allow them to contribute fully, but they are sometimes opposed by state medical societies.

### 5b) scope of practice: legal permission for semi-independent practice and to receive payment for work performed (Israel)

Circular No. 156 of the Pharmacy Division of the MoH, published at the end of 2016, defined a framework for clinical pharmacy activities in hospitals and outpatient settings, and detailed the work of the clinical pharmacist [2]. The clinical pharmacist’s activities include medication counseling and education for the medical staff and patients, as well as general activities to improve the quality of medication treatment in hospitals and communities. This includes participating in writing guidelines and protocols for using medications, creating and enforcing drug formulary policies for the institution, assessing utilization patterns, and promoting medication-related research. This circular does not address or give special permission for clinical pharmacists in performing activities relating to patient care, such as legal provision to order laboratory tests. The circular does state, however, that pharmacists with MSc or PharmD degrees should be able to prescribe with no additional training. In practice, however, this does not happen due to the complexity of the scope of practice requirements to do so.

In 2014, the Israeli Parliament updated the pharmacists’ regulation to allow a subset of pharmacists to renew medications on a very limited basis (e.g., not for more than 30 days) [15]. These pharmacists are required to have an advanced degree or to take and pass a special course, annually. The main purpose of the regulation was to prevent lapses in medication possession by patients. There was no intention to allow pharmacists the latitude to manage therapy for a chronic condition, such as diabetes, by adjusting medications. In practice, however, it has not been possible for pharmacists to do this limited sort of prescribing, due to a lack of computer infrastructure, reimbursement, and other practical considerations. To our knowledge, despite this regulation, clinical pharmacists are not currently prescribing medications in Israel.

In Israel, community pharmacists are not directly paid for direct patient care activities but rather for dispensing. Practicing clinical pharmacists are paid by their employer based on an expectation that they will improve quality and safety, but not because they bring in revenue, since there is no provision for pharmacists to bill for the work they perform.

### 6a) demand to hire clinical pharmacists by employers (United States)

Some employers in the USA have led the way in providing opportunities for meaningful clinical pharmacy practice; these employers have also led the way in employing more clinical pharmacists, and in hosting training programs for them. Among these leaders are the Veterans Health Administration (VA) and the Indian Health Service (IHS). As federal facilities, VA medical centers and IHS facilities are not subject to state law, and therefore they have historically provided an opportunity for clinical pharmacists to do more—especially in states with restrictive laws about pharmacist practice. VA medical centers also host a large proportion of the clinical pharmacy residency programs [4].

Other leaders in clinical pharmacy practice have included academic medical centers and large, group-model HMOs such as Kaiser Permanente [4]. While two decades ago, these organizations represented “islands” of clinical pharmacy practice, penetrance of clinical pharmacy into community settings (community pharmacies, community hospitals, and community physicians’ practices) is becoming more common. Given the large number of such facilities, these settings account for a very large number of jobs and contain a large community of clinical pharmacists, whose practice opportunities are continuing to increase in scope.

### 6b) demand to hire clinical pharmacists by employers (Israel)

Employers for clinical pharmacists in Israel include public hospitals (those run directly by the MoH), private hospitals such as Herzliya Medical Center, Assuta, Hadassah and Shaarei Zedek, HMO-owned hospitals such as Soroka, and several of the HMOs. A government commission recommended that the number of clinical pharmacists in public hospitals should be at least 1 per 100 beds [6]. This number is not always achieved, and even if it were, it is not sufficient to allow direct provision of care; many hospitals in the United States have considerably more clinical pharmacists than that., in addition to other supporting staff such as pharmacy technicians [16, 17].

The limiting factor for the employment of clinical pharmacists is scarce resources within HMOs, whose budgets are stretched. Therefore, each institution chooses to hire clinical pharmacists based on the individual importance that executive management finds in promoting drug therapy management and safety, in the context of considerable financial austerity.

Given their limited numbers, most clinical pharmacists in Israel work “behind the scenes,” doing appropriateness reviews and medication management counseling, writing guidelines for medication use in a hospital or HMO, promoting pharmacotherapy education among other healthcare professionals, and controlling processes such as antibiotic approvals. In private hospitals, there is no minimum number of clinical pharmacists required, and while many private hospitals do employ clinical pharmacists, it is usually at a level far lower than 1 per 100 beds, the ratio recommended for public hospitals [6].

Some of the HMOs do employ clinical pharmacists, while others do not. Clinical pharmacists are mainly found working in the central administration office of the HMOs, rather than in direct care roles. As in hospitals, their work is often administrative and consists of guideline-writing, oversight, and appropriateness reviews. Clinical pharmacists are rarely assigned directly to clinics run by the HMOs, even the large clinics. It is likely that there is a lot more work that clinical pharmacists could usefully do if there were more positions for them.

### 7a) acceptance as part of healthcare team (United States)

In the United States, previous generations of physicians had limited exposure to the field of clinical pharmacy. While many physicians trained in academic medical centers (and while 70% of US physicians have trained in a VA hospital at some point, if only for a month), older physicians trained before clinical pharmacy was as widespread as it is now. Physicians training today, however, are extremely likely to be working alongside a clinical pharmacist, and to therefore accept it as natural that the clinical pharmacist would function as a part of the healthcare team. They see the clinical pharmacist performing roles such as participating in inpatient rounds as part of the team, managing anticoagulation therapy, or accepting referrals for outpatient management of uncontrolled diabetes or hypertension. Therefore, an increasing number of US physicians (and nurses) not only accept clinical pharmacists and their role [18, 19], but frankly miss this role if they leave academic settings after residency and go to a community setting that does not have clinical pharmacy. It seems likely that in the future, their demand for clinical pharmacy to work with them will influence these settings as well.

### 7b) acceptance as part of healthcare team (Israel)

In Israel, the 2002 Circular represented important early evidence of growing widespread acceptance of and implementation of the field of clinical pharmacy [2]. Clinical pharmacy also made significant progress in 2012, with the introduction of the first graduates of the PharmD program to a number of dedicated positions opened by some of the HMOs. The active participation of clinical pharmacists in providing medication management as part of the healthcare team has been greeted with great enthusiasm by the medical and nursing staff both in the community and in the hospital setting. This is apparent in the slow but steady rise in the recruitment of additional clinical pharmacists for new positions in the community and hospitals.

Given the small number of clinical pharmacists in Israeli hospitals, they often rotate between different inpatient wards in order to expose physicians (both trainees and attending physicians) to the potential advantages of having a clinical pharmacist on the team. Anecdotally, physicians often express disappointment when the clinical pharmacist rotates off of their ward, especially when no other clinical pharmacist will be replacing them.

Further evidence of the growing esteem in which clinical pharmacists are held by other clinical staff can be found in the Report of the Committee to Improve Care on Inpatient Medicine Wards in Israel, published in 2019 [20]. This report was appointed by the director general of the MoH, and was authored by senior physicians and nurses from the HMOs, hospitals, and the MoH. One of the recommendations of the report, to improve the quality of inpatient care, was to have at least 0.25 clinical pharmacy positions for each internal medicine ward in Israel. However, these recommendations have not yet been implemented.

## Discussion

In this manuscript, we have examined seven key parameters necessary for clinical pharmacy to thrive as a field and to fully contribute to delivering high-quality care (Table 2). We contrasted the historical development and present status of these parameters between the United States and Israel. In many ways, the situation in Israel seems to be similar to that in the United States in 1960, when there were few clinical pharmacists, few pharmacists with PharmD degrees, almost no residency programs, little or no legal framework for clinical pharmacy practice in any state, and few if any jobs calling for such skills [4]. While many have lamented this state of affairs, it was our goal to examine it, to try to break it into discrete categories, and therefore to clarify exactly what changes will be needed to make progress.

**Table 2:** Comparative summary of seven key parameters to advance clinical pharmacy practice between the United States and Israel

Parameter	USA	Israel
Advanced Degree	PharmD the only degree since 2005	Small minority (1.25%) with PharmD; a similar number have an MSc degree
Postgraduate Residency	5000 residents trained each year in 2500 residency programs	No residency programs, but PharmD and MSc degrees do include some clinical components similar to a residency
Credentialing	One in eight pharmacists credentialed by BPS	No credentialing organization; no credentialed pharmacists. All pharmacists with PharmD or MSc are considered qualified to be clinical pharmacists.
Supply of Clinical Pharmacists	Over 40,000 clinical pharmacists (one per 7500 inhabitants)	Approximately 150 active clinical pharmacists (one per 60,900 inhabitants)
Legal Permission to Practice and Bill for Direct Patient Care	Varies by state, but improving. Some systems, notably VA, allow advanced practice in all locations	None (although clinical pharmacists are paid by healthcare organizations for quality improvement activities)
Employer Demand	Many employers post positions for clinical pharmacists, although others do not	Employers (HMOs and hospitals) open a small number of positions each year
Acceptance as Part of Healthcare Team	Increasing acceptance, especially with more recently trained healthcare professionals	Growing acceptance by most healthcare professionals, especially those who worked with clinical pharmacists in the past

There are reasons to expect that the situation in Israel may be about to improve. There are two schools of pharmacy in Israel (Hebrew University and BGU), and both are offering advanced degrees. These two degree programs are expected to graduate approximately 250 clinical pharmacists over the next five years. Employers, competing for these scarce and highly valued employees, can be expected to create fulfilling job positions to try to attract and retain them. It seems likely, therefore, that the situation for clinical pharmacy in Israel may be on the cusp of improving considerably – much like the situation for clinical pharmacy was on the cusp of great improvement in the United States in 1960.

Why is it important for Israel to have clinical pharmacists and to have them contribute? It is true that they contribute to quality and safety in their present roles and their present numbers, but there are many more projects that they could do that would also contribute if their numbers were greater. However, while such indirect contributions are clearly important, there are many roles that clinical pharmacists are sorely needed to fill, especially in outpatient care. Some of the tasks that clinical pharmacists perform in the United States (such as managing patients on anticoagulation therapy or with uncontrolled diabetes or hypertension) are very time-consuming tasks. Not only may clinical pharmacists do these tasks better in some cases (as measured by achieving control of the condition or preventing medication-related adverse events) [21, 22], but they also can relieve physicians of the effort and free up appointments that physicians can then use to fill roles unique to them (such as diagnosis of unknown conditions) [23]. Thus, clinical pharmacists can contribute more not only to improving quality and safety, but also to meeting currently unmet healthcare needs in Israel.

In the United States, the level of pharmacy and specifically of clinical pharmacy services has been linked with patient outcomes. A study of hospitalized patients in 855 US hospitals found that patients had lower risk-adjusted mortality when pharmacy and clinical pharmacy staffing was greater [24]. We would expect to see a similar effect in Israel, emphasizing further the importance of sufficient pharmacy and clinical pharmacy effort in the inpatient setting.

It is important to emphasize that no two countries are the same. While the United States and Israel are both OECD nations, the United States does have considerably greater resources, as measured by per capita GDP. In addition, there are cultural differences between the two countries, and this may be reflected in their values, priorities, and goals. There are important differences between the two health systems as well. The Israeli system is anchored by four large HMOs that cover all of Israel’s residents. These HMOs, plus a strong and centralized Ministry of Health, provide a highly cohesive system of health care. The United States, on the other hand, has a much less cohesive health system, with much less control by central entities and much less communication and coordination among the many entities that contribute to care delivery and financing. Given these important differences, it is unlikely that the profession of clinical pharmacy in Israel would look just like it does in the United States, even if we were to imagine that it could be fully “realized” as a profession.

It is also important to mention that even the United States has not fully “arrived” in terms of clinical pharmacy roles. While the United States has in many ways led the development of this profession, clinical pharmacists continue to push for a greater role. Even in the VA system, clinical pharmacists have increased their role greatly in just the past 5 years [25]. This reminds us of how recent the development of clinical pharmacy is, and how it is in many ways still incomplete.

### Policy recommendations: Israel

The impetus for this article was to explore differences between the United States and Israel regarding the current status of clinical pharmacy, and to draw lessons from this about how Israel can progress. Based on the comparison made in this manuscript, we recommend the following concrete steps to advance the field of clinical pharmacy in Israel:

- Clinical pharmacy should be recognized as a specialty within pharmacy. It would be logical for the MoH to lead this change, by mandating that clinical pharmacy should be a special subset of pharmacy, just like nurse practitioners are a special subset of nurses. This change would contribute to the creation of more positions specifically intended for clinical pharmacists, greater opportunities for professional promotion, and improved wages. All of these, in turn, would be expected to attract more pharmacists to acquire higher education in clinical pharmacy.
- The MoH, together with pharmacist organizations, should facilitate the creation of a professional body, including input from academia, to define what curricular elements should be included in Israeli postgraduate pharmacy residency programs. Once residency programs have been established, the committee or its successor would oversee residency programs for clinical pharmacists, as ASHP does in the United States today. Residency programs, once created, would provide added value to trainees, to the institutions that train them, and to the institutions that employ them after program completion.
- Efforts should be made to expand the activities of clinical pharmacists beyond counseling patients about medication use. Expanded activities should include writing medication orders under treatment protocol and/or under the supervision of a physician, ordering laboratory tests relevant to treatment, and taking a medication-related history. This change would involve changes in legal and regulatory frameworks, as well as specific encouragement by employers for clinical pharmacists to pursue these activities.
- More patients should receive consultation from clinical pharmacists, especially as part of ambulatory care provided by HMOs or as an additional paid service. This would be especially helpful for patients with multiple comorbidities or those taking multiple medications, and would contribute to better outcomes for patients and also would likely save money through averted adverse events.
- There is a need to reevaluate the number of clinical pharmacists required in the hospital, both in public and private hospitals, as well as a need to enforce whatever standard is agreed to. Without a predefined standard, it will be impossible to fully realize the vision of clinical pharmacy. Hospital based clinical pharmacists are especially needed in internal medicine, surgery, intensive care, oncology, geriatrics and pediatric wards, where polypharmacy is prevalent or where there is a high potential for medication errors.
- While Israel has a recommended minimal level of clinical pharmacy staffing in public hospitals, there is no defined a minimal level of staffing in outpatient settings. This too should be done.
- Integrating clinical pharmacist effort in an antibiotic stewardship program, such as exists at most hospitals, is highly recommended as part of infection control efforts.
- Pharmacists should take a more central role in the medication reconciliation processes during transition from hospitalization to the community, and vice versa, in order to prevent common drug errors. This would require dedicated effort on the part of many clinical pharmacists, but would also provide great value.
- While Israeli law allows for limited prescription of medication by clinical pharmacists, mainly to avoid lapses in medication possession, even this limited goal has not been realized because the necessary infrastructure to support it has been lacking.
- Progress has been made over the last few years with the MoH publications dealing with clinical pharmacy and providing medication-related consultation to patients. The next step is to find the resources to implement those ideas into practice. It is the responsibility of the MoH and the HMOs to more fully utilize clinical pharmacists as part of their commitment to patient health as enshrined in Israel’s Health Insurance Law of 1995 [26]. Israel’s State Comptroller also gave similar recommendations as part of a recent report regarding regulations for pharmacy supervision in Israel [27].

## Conclusions

While Israel’s pathway to full realization of clinical pharmacy as a profession may not look precisely the same as it did in the United States, our comparative analysis suggests that one would need to go back 60 years in the United States to find a parallel to the current situation in Israel. If clinical pharmacy is to maximize its contribution to improving the quality of care in Israel, and also contribute to addressing the urgent shortage of healthcare personnel in Israel, many changes will need to occur, as per the policy recommendations we outlined above. Our hope is that this manuscript, by delineating and analyzing seven key parameters together with our concrete policy recommendations, will give a better sense of just what would have to change, and how.

## Data Availability

Not applicable.
